# Designing Conversational Agents to Support Self-Regulated Learning for Older Adults

**DOI:** 10.1093/geroni/igab046.476

**Published:** 2021-12-17

**Authors:** Jessie Chin, Smit Desai

**Affiliations:** 1 University of Illinois at Urbana-Champaign, University of Illinois at Urbana-Champaign, Illinois, United States; 2 University of Illinois, Urbana Champaign, Champaign, Illinois, United States

## Abstract

The rapid growth of the off-the-shelf smart speakers (such as Amazon Alexa and Google Home), also called Conversational Agents (CAs), creates potential to deliver everyday life support to users at home (such as checking weather, listening to news, scheduling events). Literature demonstrated the technology acceptance of CAs among older adults (including novice users) given the low barriers to use CAs. The natural conversations among CAs and users enable the opportunities to build deeper understandings about a topic through theory-driven guided dialogues. Our study has designed the metacognition strategies in the guided dialogues of CAs to support informal self-regulated learning of health information among older adults. The study has shown the feasibility and acceptance of CAs to help older adults learn new health information on their own through these guided dialogues. Additional analyses on the feasibilities to implement different metacognitive strategies in guided dialogues in the off-the-shelf CAs were also conducted.

